# Regulation of gene-edited plants in Europe: from the valley of tears into the shining sun?

**DOI:** 10.1007/s42994-023-00130-8

**Published:** 2023-12-28

**Authors:** Holger Puchta

**Affiliations:** Department of Molecular Biology, Joseph Gottlieb Kölreuter Institute for Plant Sciences, Karlsruhe Institute of Technology (KIT), Fritz-Haber-Weg 4, 76131 Karlsruhe, Germany

**Keywords:** Genetically modified organism, Cisgenesis, Gene editing, CRISPR/Cas, Regulation

## Abstract

Some 20 years ago, the EU introduced complex regulatory rules for the growth of transgenic crops, which resulted in a de facto ban to grow these plants in fields within most European countries. With the rise of novel genome editing technologies, it has become possible to improve crops genetically in a directed way without the need for incorporation of foreign genes. Unfortunately, in 2018, the European Court of Justice ruled that such gene-edited plants are to be regulated like transgenic plants. Since then, European scientists and breeders have challenged this decision and requested a revision of this outdated law. Finally, after 5 years, the European Commission has now published a proposal on how, in the future, to regulate crops produced by new breeding technologies. The proposal tries to find a balance between the different interest groups in Europe. On one side, genetically modified plants, which cannot be discerned from their natural counterparts, will exclusively be used for food and feed and are—besides a registration step—not to be regulated at all. On the other side, plants expressing herbicide resistance are to be excluded from this regulation, a concession to the strong environmental associations and NGOs in Europe. Moreover, edited crops are to be excluded from organic farming to protect the business interests of the strong organic sector in Europe. Nevertheless, if this law passes European parliament and council, unchanged, it will present a big step forward toward establishing a more sustainable European agricultural system. Thus, it might soon be possible to develop and grow crops that are more adapted to global warming and whose cultivation will require lower amounts of pesticides. However, there is still a long way to go until the law is passed. Too often, the storm of arguments raised by the opponents, based on irrational fears of mutations and a naive understanding of nature, has fallen on fruitful ground in Europe.

## Introduction

Mutation and selection are not only the principle of natural evolution, but are also the basis of classical plant breeding. Due to naturally occurring mutations, all individuals of a crop grown in the same field differ genetically from each other. Taking into account the genome size, one would expect to find at least 100 mutations per individual in barley, according to conservative estimations (Ossowski et al. 2010). Moreover, it turns out that, beside point mutations and changes induced by transposable elements (Sharma and Peterson 2023), structural variations, such as chromosomal inversions (Zhou et al. 2023; Crow et al. 2020), also occur regularly in the gene pool of a plant species. Occasionally, these rare mutations can result in advantageous traits for farming. In fact, for millennia, breeders were able to develop most of the crops that are grown on our fields today. In the twentieth century, it became clear that the mutation rates are not invariable, but can be influenced by specific treatments. Using gamma rays or genotoxic chemicals, the occurrence of phenotypic mutations could be greatly increased. Thus, this rate-limiting step in the breeding process could be overcome and it became possible to obtain much more attractive traits for farming in a much shorter time frame (Parry et al. 2009; Programme 2009). In this way, short-stemmed barley and durum wheat could be obtained—and we benefit from this technical advance every day when drinking a beer or eating pasta (Braumann et al. 2018; Xiong et al. 2018).

However, using classical mutagenesis comes with a price: thousands of off-site mutations are induced at the same time (Li et al. 2016). Although most of them will likely not result in phenotypes, often plants arise that also acquire, besides the agronomically beneficial novel trait, further changes that have adverse effects on growth or yield. Thus, in multiple rounds of back-crossing with the original cultivar, the advantageous trait must be genetically separated from other unwanted phenotypic mutations. This process can take several years. Moreover, it is not possible to eliminate undesired mutations if they lie in closer proximity to the advantageous trait on the same chromosome. Nevertheless, this technique proved to be very successful and thousands of cultivars have been obtained that are in use, worldwide, thanks to classical mutagenesis (Programme 2009). However, one has to keep in mind that such “cured” plants carry not only the beneficial trait, but also hundreds to thousands of off-site mutations within their genome.

## The current EU regulation of plant GMOs

With the rise of plant transformation at the end of the last century, it became possible to obtain crops with novel traits by the integration of foreign genes. However, with the advent of this new technology, also new questions arose, such as, whether these transgenic plants would be safe for consumers and the environment and how they should be regulated. Taking the precautionary principle into account, the European Union introduced the GMO directive 2001/18/EC (https://eur-lex.europa.eu/eli/dir/2001/18/oj), which is still in place 20 years later. Unfortunately, the precautionary principle used by the EU focused only on putative risks. Clearly, it was a gross misunderstanding of this principle to not consider the potential positive impacts. The respective regulation requires complex and long-term evaluations before GMOs can be planted in the field. Thus, only global players in the breeding industry, which were willing to invest large sums of money, could elect to use this option. In practice, hardly any GMOs were grown in the EU, and in many countries, no GMOs at all were released into their agricultural systems. In public discussion, the use of GMOs, and especially the business practices of companies like Monsanto, was commented on very critically. This went as far as the violent destruction of fields in which GMOs were grown for research purposes by militant opponents. Very promising approaches, developed in Europe to solve problems of nutritional deficiencies, such as the development of golden rice by Ingo Potrykus and Peter Beyer, were demonized by NGOs (Ye et al. 2000).

An important factor underlying EU compliance regulation, in terms of implementation and control, was the fact that foreign genes could easily be detected by various molecular techniques in the genomes of the respective crops. Thus, mostly by a simple PCR assay, the presence or absence of a specific transgene could be monitored. It also became possible to detect transgenic contaminations in grain imports into the EU. As a consequence, in April 2004, an arbitrary threshold value was set in the EU for food and feed by the EU directive 1829/2003 (http://data.europa.eu/eli/reg/2003/1829/oj): if 0.9% or more of the grains of the respective sample contained a transgene, the import into the EU was banned.

Additionally, taking the precaution principle into account, several government- and EU-funded research projects were initiated to analyze, in detail, all possible risks that transgenic crops might cause for consumers or the environment. These extensive studies—in Germany alone they carried out over the course of 25 years—did not produce any indication that crops carrying transgenes are more dangerous than conventionally produced crops in any shape or form (Kessler 2001; Deutschland Bundesministerium für Bildung und Forschung and Minol 2014).

Although the induction of genetic modifications by the use of site-specific nucleases was already shown to be achievable in plants, some 30 years ago (Puchta 2016), it took a long time to develop efficient programmable enzymes that could be used to induce changes beneficial for breeding in the crop genome. This was fist achieved by the use of zinc finger nucleases (Shukla et al. 2009; Townsend et al. 2009) and later by using TALENs (Zhang et al. 2013). Nevertheless, only with the discovery of Cas9, as an efficient and easily programmable nuclease (Jinek et al. 2014), was it possible to induce mutations in any gene of interest, as desired. In a major breakthrough, powdery mildew-resistant wheat was obtained by TALENs- and CRISPR/Cas-mediated induction of mutations in the three *MLO* genes (Wang et al. 2014). This was possible because it was discovered that a mutation in this gene could lead to a natural powdery mildew resistance in barley. This example nicely illustrates the power of this new technology: if a natural mutation is characterized in a specific crop that then leads to an advantageous trait, it is possible to improve other crops, agronomically, very quickly and independently of species borders, by the directed induction of the analogous mutation in the respective homolog. Moreover, induction of genetic resistances against pathogens will lead to a dramatic reduction in pesticide use and, thus, to a more sustainable agriculture. In this regard, one of the central demands of ecologists and NGOs, in Europe, is that the use of pesticides in agriculture needs soon to be drastically reduced.

Taking the natural mutation frequency and the fact that the new technology only induces single mutations at unique positions into the genome into account, it became clear that plants with changes obtained by editing could not be discerned from untreated plants. This applies to all nucleases that induce double-stranded breaks (DSBs), if the genes coding for the editing tools are either not integrated or segregated out after the site-directed mutation has been introduced. DSBs also occur naturally, not only during replication and transposition, but can also be induced by natural irradiation. In all of these cases, DSBs are repaired by the cellular repair machinery: mainly by non-homologous end joining, which often leads to small indels (Puchta 2005). The same kind of mutation pattern arises independently from the cause of the DSB. The same holds true for base editing (Komor et al. 2016), as point mutations, due to the incorporation of the wrong bases during replication or due to the deamination of bases, occur regularly.

Thus, by analyzing a plant, it is not possible to define whether a specific change was introduced by editing or has occurred naturally (Grohmann et al. 2019; Broll et al. 2019). Therefore, the general expectation of the EU scientific and breeder community was that these plants would be exempt from the GMO regulation—just as plants obtained by classical mutagenesis. In contrast to classical mutagenesis, CRISPR nucleases induce few—if any—off-site mutations (Tang et al. 2018), whereas thousands of mutations are present in plants obtained by classical mutagenesis. Therefore, the new technology would present a tremendous advance, also in relation to biosafety.

In 2018, the European court of justice was called to decide on this matter, taking the EU regulation of 2001 into account. To the big surprise of most observers, the judgment did not take scientific arguments into account, but adhered to the old regulation in a literal way (https://curia.europa.eu/juris/liste.jsf?language=en&td=ALL&num=C-528/16). The basis for this decision was that, in the end, not the product (even if it is identical to a natural plant), but the process of how it was obtained is legally decisive for the classification, according to the existing law. Thus, the use of CRISPR/Cas—like the use of classical mutagenesis—was classified as an artificial way of achieving genetic change and, therefore, plants obtained with this technology had to be regarded as GMOs, and regulated accordingly. However, since classical mutagenesis had been widely used in breeding for more than half a century, without adverse effect, the court suggested that, due to this long safety record, the member states would be able to exempt these plants from regulation (Eriksson 2019). Nevertheless, it was not defined by the court as to how such a “history of safe use” can be achieved.

The European scientific community was shocked by this judgment as, in practice, it meant that plants with genetic changes, induced in a “shotgun way”, were allowed to be grown on any field, whereas crops with sophisticated scalpel-like changes were practically forbidden to be grown in the EU. As a consequence, funding agencies, such as the German Ministry of Science, phased out research grants on plant gene editing and current are not initiating any new ones. Due to the lack of appropriate academic and industry jobs for young scientists, a quite possibly irreversible brain and skill drain has been initiated from the EU to other countries, such as the USA. European breeding companies relocated their gene editing research units to the USA. Moreover, the hope that innovative new approaches could be developed in the field of plant gene editing in Europe, via the foundation of startup companies, was blocked once and for all due to the immense regulation cost that would have to be carried by such companies. Thus, although European groups contributed significantly to the development of plant gene editing technologies (Boch et al. 2009; Puchta et al. 1993), in recent years, it became increasingly more difficult for them to keep pace with the impressive advances that have been achieved in the USA and China. Thus, in contrast to the USA and China, where industry invested heavily, as did government programs, on gene editing approaches, the recent years of stagnation in Europe resulted in quite a massive loss of competitiveness in this research area.

To change the situation, a number of initiatives were started by scientists to convince politicians, and also the general public, that the current situation was not only scientifically unbearable, but also harmful for European agriculture. The European Sustainable Agriculture through Genome Editing network (EU SAGE) organization was launched, bringing together European research institutes as well as individual scientists (Dima and Inzé 2021). A number of scientific academies, such as the German Leopoldina, published statements (German National Academy of Sciences Leopoldina et al. 2019) to convince the public as well as politicians that a “scientifically justified, differentiated regulation of genome edited plants” should be established in the EU.

## The suggestion of the EU Commission

To prepare a change in legislation, the European Commission initiated discussions with all interest groups and started a public online consultation. Finally, in July 2023, they presented their suggestion for a new law, regulating the “New Genomic Techniques” (NGT) as part of their new strategies “Green Deal” and “Farm to Fork” to increase the sustainability and resilience of the European food system (https://food.ec.europa.eu/plants/genetically-modified-organisms/new-techniques-biotechnology_en#commission-proposal-on-plants-obtained-by-certain-new-genomic-techniques). Interestingly, the definition of NGT not only included the use of editing technologies, but also the use of cisgenesis. This allows the use and integration of sequences that are already present at other sites and in other combinations in the genome of the respective organisms or the gene pools of sexually crossable plants.

The rationale behind the suggestion is that NGT plants, which could also occur naturally or could be produced by conventional breeding, should not be regulated as GMOs anymore. Also, their products should not have to carry any label. However, for the EU Commission, it matters for what kind of purpose these plants are produced. If they are used for food or feed, they must be registered in a public database, but otherwise are exempt from any further regulation. In an Annex belonging to the suggested law, the different kinds of traits obtained by NGT are listed, which the Commission regards as relevant for a food and feed classification (Annex III, see Table 1). According to this list, every genetic change that helps to decrease biotic or abiotic stresses, as well as every change that decreases the necessary input in water, fertilizer, and pesticides, or increases the yield or the quality of the resulting products, should not be regulated. In contrast, if the edited trait is herbicide resistance, the plant will not be exempt from regulation. In these cases, the same, extensive, kind of risk assessment has to be performed as for GMOs. Thus, it is not likely that the use of herbicides will be promoted by opening the European market for NGT plants. In fact, a massive reduction in the use of herbicides, pesticides and fertilizers in the EU is an aim that the Commission and European NGOs agree on. By applying NGT, it will be possible to introduce genetically based resistances to protect future harvests much better from pathogen infections and, thus, reduce pesticide use. Also, if NGT-modified plants become more resistant to abiotic stresses, such as heat or drought, in the long run farmers will need less fertilizer and water than with conventional crops to safeguard yields.

**Table 1:** Plant traits obtained by NGT that will not be regulated, as defined in Annex III

Traits justifying the incentives referred to in article 22 of the suggested law	
1	Yield, including yield stability and yield under low-input conditions
2	Tolerance/resistance to biotic stresses, including plant diseases caused by nematodes, fungi, bacteria, viruses and other pests
3	Tolerance/resistance to abiotic stresses, including those created or exacerbated by climate change
4	More efficient use of resources, such as water and nutrients
5	Characteristics that enhance the sustainability of storage, processing and distribution
6	Improved quality or nutritional characteristics
7	Reduced need for external inputs, such as plant protection products and fertilizers

Like the NGOs, the organic food production industry plays an important role in European politics and economy, having considerable influence on consumers as well as parliamentarians. A central dogma of the organic industry is the complete exclusion of GMOs from the food production chain. As edited plants are regarded as GMOs, by these organizations, it is essential for their business model to be able to actively exclude such plants from their fields. The commission’s suggestion respected this interest by prohibiting the use of NGT plants for organic production. This is possible due to the fact that all plants obtained by NGT have to be listed in a public register. Thus, organic farmers are indeed able to exclude all kinds of transgenic or gene-edited plants from their production. Thus, although once and again it was claimed that permitting gene-edited plants to the European market would ruin organic farmers, the suggestion of the commission safeguards that they will be able to act according to their business model. Likewise, all consumers that do not wish to eat food produced from gene-edited crops will be able to so by buying European organic food. Thus, the suggestion of the commission also takes care of the demand of the NGOs to guarantee consumer choice.

## What kind of directed genomic changes might be allowed on European fields in future?

With the suggested law, the commission redirects the present classification strategy from a process to products. As demanded by the scientific community, it is now the genetic change in the product that matters, not the method used to achieve the genetic change. From the scientific viewpoint, another very interesting question to be answered is which exact types of modification will be regarded by the European Commission as being equivalent to naturally occurring mutations. Indeed, it is not trivial to define which changes can be regarded as being a natural-identical genomic change(s) over time; the use of more sophisticated genome analysis technologies have led to the discovery of more and different kinds of naturally occurring variations (Qin et al. 2021; Jayakodi et al. 2020; Zhou et al. 2023). The respective named criteria, which are defined in the Annex I of the proposal, are depicted in Table 2. It is remarkable that these different kinds of changes were not only defined, but they were, at least partially, also quantified.

**Table 2:** Criteria for the equivalence of NGT plants to conventional plants, according to Annex I of the proposal for a new regulation of the European Commission

An NGT plant is considered equivalent to conventional plants when it differs from the recipient/parental plant by no more than 20 genetic modifications, of the types referred to in points 1–5, in any DNA sequence sharing sequence similarity with the targeted site that can be predicted by bioinformatic tools	
1	Substitution or insertion of no more than 20 nucleotides
2	Deletion of any number of nucleotides
3	On the condition that the genetic modification does not interrupt an endogenous gene: (a) targeted insertion of a contiguous DNA sequence existing in the breeder’s gene pool (b) targeted substitution of an endogenous DNA sequence with a contiguous DNA sequence existing in the breeder’s gene pool
4	Targeted inversion of a sequence of any number of nucleotides
5	Any other targeted modification of any size, on the condition that the resulting DNA sequences already occur (possibly with modifications as accepted under points (1) and/or (2)) in a species from the breeders’ gene pool

Five classes of mutations are listed, with the first being insertions/substitutions of up to 20 nucleotides. However, how long does a consecutive sequence need to be for it to statistically be identified as foreign? Relevant for the answer is of course the size of the host genome: the larger the genome, the higher is the probability that any combination of bps of a certain length can be found in the respective genome. Taking large crop genomes into account, such as *Vicia faba* with more than 13 billion bp (Jayakodi et al. 2023), a sequence below 20 bps length cannot be statistically identified as foreign. Thus, although seemingly arbitrary, at first sight, this classification is not only scientifically explainable, but also legally justified.

As the second class, deletions of any size are listed. Obviously, besides point mutations, deletions are the most common class of natural mutations. They occur very often during DNA replication or the activation of transposable elements, but can also be induced by radiation.

The third class covers the possibility of introducing cisgenic sequences. The principle of cisgenesis relies on the idea that only sequences that are already present in the genome of organism are introduced in a novel combination at a different genomic site for trait improvement (Hou et al. 2014; Holme et al. 2013). Thus, no foreign “trans” gene is inserted into the organism. The EU commission suggests that it should be possible to use all sequences of the gene pool of the respective species, as long as their insertion or replacement does not interrupt an endogenous gene. This is remarkable and farsighted, as cisgenesis is not regulated in the same way in other countries. Its inclusion should permit diverse modifications in gene expression and dosage, as well as the introduction of novel traits from genes of crossable varieties.

As the fourth class, inversions of any size are listed. Only in recent years, due to the use of long read sequencing, it has become clear that inversions often occur naturally during genome evolution and are found regularly in different crop cultivars (Zhou et al. 2023; Jayakodi et al. 2020). As a consequence, within the inverted region, no genetic exchange takes place. Recent progress in genome editing technologies has made it possible to engineer entire plant chromosomes (Rönspies et al. 2021). In a proof of concept experiment, it could be shown that an Mbp-sized inversion, which had occurred 5000 years ago in *Arabidopsis* and led to suppression of crossover formation in the respective region between different ecotypes, could be reversed to bring back genetic exchange (Schmidt et al. 2020). Beside unlocking regions of the genome for meiotic recombination, inversions can also be used to link traits and protect them from segregation. By introducing an inversion of almost the entire chromosome 2 of *Arabidopsis*, crossovers could be almost completely suppressed in 1/8th of the *Arabidopsis* genome (Rönspies et al. 2022). The induction of a large inversion has also been achieved in corn (Schwartz et al. 2020) and rice (Lu et al. 2021). Besides redirecting genetic exchange, inversions can also be used to exchange promoters between genes to modulate their expression (Lu et al. 2021). Thus, the exemption of inversions from regulation, as a specific class of natural mutations, is not only scientifically justified, but also very farsighted.

As a fifth class, all sequences that are available in the gene pool of the respective crop species can be introduced into the respective cultivar without any size limit. Thus, even plants containing sequences differing in much more than 20 bps do not have to be regulated, if these sequences can be found in the gene pool of the respective species. As no size limitation has been defined, this should also include chromosomal substitutions, additions or translocations (Beying et al. 2020), which can naturally arise during plant genome evolution (Mandáková and Lysak 2018), especially following polyploidizations. Moreover, recent long read sequencing experiments revealed that there exist quite large variations between individual cultivars of cereals and that their genomes differ in content and size by up to several percent (Jayakodi et al. 2020; Qin et al. 2021; Zhou et al. 2023). The sum of these variations is defined as the pan-genome of a species, which contains the sum of genetic information that is present in some, but not all, individuals of a species. As all sequences present in the pan-genome could also be transferred into the recipient, by classical crossing, it is obvious that introducing such sequences should be exempt from regulation.

Finally, plants are considered equivalent to conventional plants when they differ from the recipient/parental plant by no more than 20 genetic modifications, as defined in the five classes above. Thus, plants obtained from multiplexing, the simultaneous editing of several genes for complex trait improvements, will not be regulated (Zhou et al. 2019). This also opens the door for breeders to perform the de novo domestication of wild species carrying abiotic stress or pathogen resistance genes (Curtin et al. 2022): in these cases, the simultaneous induction of mutations in several individual genes is required. First, examples of de novo domestication have already been achieved with wild relatives of tomato, physalis and rice (Yu et al. 2021; Zsögön et al. 2018; Li et al. 2018). However, as some crops are polyploid, it is necessary to induce the same kind of change in each individual haploid chromosome set. Thus, in hexaploid wheat, six individual copies of a gene have to be mutated to achieve a functional knockout. Therefore, the number of changes given in Annex 1 should be specified to 20 genetic modifications, per haploid genome. Of course, one could argue about whether, or not, the number of 20 changes, or 20 mutations, is rational when classical mutagenesis results in several thousands of mutations per genome. Nevertheless, the quality and the quantity of the introduced natural-identical mutations, described in Annex 1, are scientifically sound and farsighted. Due to global warming, Europe is in urgent need for crop plants that are more heat and salt resistant/tolerant and require less pesticide use. The rules suggested by the EU Commission open the door for growing such plants in Europe, which will ultimately result in the establishment of a more sustainable agricultural systems.

## Conclusion

It would seem to be easier to square a circle than to balance the interests of the different players when it comes to defining a new regulation for gene-edited crops in Europe. Therefore, the proposal of the Commission is, in a way, a very Solomonic solution to untangle this Gordian knot. Most importantly, the Commission took up its responsibility to pave the way for a more sustainable agricultural system in Europe in times of global warning. At the same time, it took concerns regarding herbicide use in the EU by environmentalists into consideration and guarantees the survival of the European organic sector by banning gene-edited plants from use in organic farming thereby guaranteeing consumer choice.

In comparison to regulations in other countries, the suggested list of genetic modifications that will not be regulated in Europe is more progressive and farsighted, especially in respect to inversions as well as cisgenic sequences. This could prove as very helpful for European breeders to catch up with the rest of the world. However, the proposal does not address issues of intellectual property, which remains an open question that the commission has to, and will, address in the next years. Moreover, it is still a long way to go before the current suggestion becomes a law: after a further round of public consultation, the text might be subjected to changes. Indeed, a storm of arguments was raised by the opponents after the suggestion had been published. Their line of argumentation is mainly based on irrational fears of mutations and a naive understanding of nature and agriculture. Unfortunately, despite being scientifically questionable, these arguments have all too often fallen upon fruitful ground in Europe. Finally, the suggested law has to be passed by the European Parliament and the European Council which could result in further changes, along with potential further delays. As the European Parliament will be reelected in June 2024, the suggestion will most likely only enter the legislation process in the autumn of 2024. Nevertheless, with the balanced suggestion of the Commission, the first important step has been made, although it might still take years before we will see the first gene-edited crops being grown in European fields.
